# *De novo* Transcriptome Assembly of Chinese Kale and Global Expression Analysis of Genes Involved in Glucosinolate Metabolism in Multiple Tissues

**DOI:** 10.3389/fpls.2017.00092

**Published:** 2017-02-08

**Authors:** Shuanghua Wu, Jianjun Lei, Guoju Chen, Hancai Chen, Bihao Cao, Changming Chen

**Affiliations:** ^1^Department of Vegetable Science, College of Horticulture, South China Agricultural UniversityGuangzhou, China; ^2^Vegetable Research Institute, Guangdong Academy of Agricultural SciencesGuangzhou, China

**Keywords:** Chinese kale, *de novo* assembly, RNA-seq, transcriptome, glucosinolate metabolic pathways, gene expression, multiple tissues

## Abstract

Chinese kale, a vegetable of the cruciferous family, is a popular crop in southern China and Southeast Asia due to its high glucosinolate content and nutritional qualities. However, there is little research on the molecular genetics and genes involved in glucosinolate metabolism and its regulation in Chinese kale. In this study, we sequenced and characterized the transcriptomes and expression profiles of genes expressed in 11 tissues of Chinese kale. A total of 216 million 150-bp clean reads were generated using RNA-sequencing technology. From the sequences, 98,180 unigenes were assembled for the whole plant, and 49,582~98,423 unigenes were assembled for each tissue. Blast analysis indicated that a total of 80,688 (82.18%) unigenes exhibited similarity to known proteins. The functional annotation and classification tools used in this study suggested that genes principally expressed in Chinese kale, were mostly involved in fundamental processes, such as cellular and molecular functions, the signal transduction, and biosynthesis of secondary metabolites. The expression levels of all unigenes were analyzed in various tissues of Chinese kale. A large number of candidate genes involved in glucosinolate metabolism and its regulation were identified, and the expression patterns of these genes were analyzed. We found that most of the genes involved in glucosinolate biosynthesis were highly expressed in the root, petiole, and in senescent leaves. The expression patterns of ten glucosinolate biosynthetic genes from RNA-seq were validated by quantitative RT-PCR in different tissues. These results provided an initial and global overview of Chinese kale gene functions and expression activities in different tissues.

## Introduction

Chinese kale (*Brassica oleracea* var. *alboglabra* Bailey) is native Chinese brassica vegetable which is widely distributed in Southern China and Southeast Asia (Qian et al., 2016). Besides good flavor, the stem and leaves are high in anticarcinogenic compounds and antioxidants, including vitamin C, total phenolics, carotenoids, and glucosinolates (Sun et al., 2011a,b, 2012).

Glucosinolates are a group of secondary metabolites containing nitrogen and sulfur, mainly found in the order Capparales. They play important roles in plant defense and in human nutrition (Fahey and Talalay, 1999; Halkier and Gershenzon, 2006). It has been clinically proven that some glucosinolate-derived isothiocyanate and nitrile compounds display anticarcinogenic activity (Fahey and Talalay, 1999; Halkier and Gershenzon, 2006). To date, the variations in glucosinolate content and profile were observed in most of the brassica plants in different organs, cultivars, growth stages, and growing conditions (Xu et al., 2010; Yuan et al., 2010; Sun et al., 2011a,b, 2012; Lee et al., 2013, 2014; Zhu et al., 2013; Bhandari et al., 2015; Yi et al., 2015). The contents and profile of glcosinolate in different tissues or organs of Chinese kale was previously reported (Sun et al., 2011a,b, 2012). Depending on the amino acids from which they are synthesized, glucosinolates are divided into three major groups: aliphatic, indolyl, and aromatic glucosinolates (Halkier and Gershenzon, 2006). Generally, the biosynthesis of glucosinolate occurs via three separate phases: the chain elongation of precursor amino acids, the formation of the core structure, and modification of the side chain of glucosinolate (Sønderby et al., 2010). A number of key regulators and genes involved in the biosynthetic network of glucosinolate that present in the genus *Arabidopsis* are known (Grubb and Abel, 2006; Sønderby et al., 2010). From that basis, the genes involved in glucosinolate metabolism were identified in many plants, such as *Brassica rapa* (Zang et al., 2009; Wang H. et al., 2011), Cabbage (*B. oleracea* sp. Capitata, Liu et al., 2014), Broccoli (*B. oleracea* var. italic, Gao et al., 2014), and radish (Wang et al., 2013). High glucosinolate content is a main trait of Chinese kale cultivars; it mediates nutritional quality and flavor in the stem and leaves (Sun et al., 2012). Previous studies mainly focused on determining variation in the composition or content of glucosinolate in different cultivars, organs, growing conditions, and growth stages of Chinese kale (Sun et al., 2011a,b, 2012; Qian et al., 2015, 2016). Although, there were studies focused on *AOP2* gene function analysis, and expression analysis of some glucosinolate biosynthetic genes (Qian et al., 2015; Yin et al., 2015), there have been no comprehensive reports of genes involved in glucosinolate metabolism and its regulation in Chinese kale and no reports on these gene expression patterns in different tissues.

To date, genome resources consisted of Brassica plants, such as *Brassica napus* (Chalhoub et al., 2014), *B. rapa* (Zang et al., 2009; Wang X. et al., 2011), *B. oleracea* sp. Capitata (Liu et al., 2014), and Broccoli (Gao et al., 2014). However, there were no publications on RNA-seq analysis of Chinese kale, and very few reports of RNA-seq for different tissues of *Brassica* plants. In this study, we sequenced and characterized the transcriptomes and the expression profiles of genes expressed in 11 different tissues of Chinese kale using high-throughput sequencing technologies (Nagalakshmi et al., 2010). From the gene sequence reads and expression data, we assembled comprehensive unigene sets for Chinese kale, functionally categorized them *in silico*, and identified their expression levels and co-expression networks in 11 tissues. For the first time, we also identified the genes involved in glucosinolate biosynthesis and regulation, and analyzed their expression patterns in different tissues. Therefore, the results and findings of this study provide an initial and global overview of Chinese kale gene functions and expression activities in different tissues.

## Materials and methods

### Plant materials and preparation

The seeds of Chinese kale (*B. oleracea* var. alboglabra Bailey cv. Zhonghua) were germinated in plastic pots. The seedlings were cultured in the field at 22–25°C at South China Agricultural University (Guangzhou, China). After 3 weeks, the seedlings with 4 true leaves were transplanted into a greenhouse field to avoid rainwater. Water, fertilizer, and pesticides were applied as necessary (Qian et al., 2015; Yin et al., 2015). When the plants were fully grown, samples for RNA extraction were harvested free of any insects and mechanical damage (Supplementary Image 1, Qian et al., 2015; Yin et al., 2015). The chlorophyll content in leaves was about 1500 μg·g^−1^ fresh weight and the inflorescence was as high as the apical leaves at this stage (Sun et al., 2011a,b; Qian et al., 2015; Yin et al., 2015). The plants were divided into 11 parts (further called “tissues” in this article) as shown in Supplementary Image 1. All samples were cleaned and washed with double distilled water. Pieces of tissues from more than 5 plants were pooled into one sample. All tissue samples were frozen in liquid nitrogen immediately after collection and stored at −80°C until further analysis.

### RNA extraction, library construction, and RNA-Seq

A total of 100 mg of frozen samples of each tissue were ground into fine powders in liquid nitrogen and total RNA was extracted using TRIzol reagents (Invitrogen, USA), all according to the manufacturer's instructions. Each RNA sample was subjected to DNase digestion (TaKaRa, Dalian, China) to remove any remaining DNA. The RNA content was quantified by spectrophotometry (BioPhotometer plus, Eppendoff, Germany) and checked on 1.2% denaturing agarose gels. The RIN (RNA integrity number) values (>8.0) of these samples were assessed using an Agilent 2100 Bioanalyzer (Agilent Technologies, Santa Clara, CA, USA). The construction of the libraries and the RNA-Seq were performed by the Biomarker Biotechnology Corporation (Beijing, China). All procedures for cDNA library construction were performed via the standard Illumina sample preparation protocol. Sequencing of the purified libraries was carried out on an Illumina HiSeq 4000 platform (Illumina, Inc., USA), with one lane of 2 × 151 bp reads from both ends of the fragments (“paired ends”). In the present study, the biological replications should have been made, but we performed qRT-PCR to verify and confirm the results of RNA-seq. Although, RNA-seq results will be better with biological replicates, it has been shown earlier that biological replicates might be not necessary for digital gene expression profiling by high-throughput RNA sequencing (Wang K. et al., 2015).

### Quality control and *de novo* construction of transcripts

Raw reads in fastq format were first processed by Trimmomatic (Bolger et al., 2014). In this step, high quality reads were achieved by removing the reads containing an adapter, reads containing ploy-N and low quality reads from raw data. At the same time, Q20, Q30, and GC contents were calculated. The Bwa (Li and Durbin, 2009) was employed to align all high quality reads against the silva database (Pruesse et al., 2007). After this, reads of rRNA were removed and clean reads were obtained. Reads of each tissue and the whole plant were then *de novo* assembled into gene sets separately using Trinity and CD-hit (Grabherr et al., 2011; Fu et al., 2012). Gene sets from each tissue were used for tissues transcriptome comparison. Genes that were constitutively low expressing (FPKM<1 calculated, Davidson and Oshlack, 2014) in all tissue were filtered from the gene set of the whole plant, which served as the reference gene set in the following analysis.

### Functional annotation and classification

All genes were aligned against public databases (Nt, Nr, COG, Swissprot, KEGG) to obtain their putative function leveraging blast+ with *e* <= 1e-5 and query coverage >= 0.33. Based on Nr annotation, a species list of all best hit (highest bit scores in all alignments) was extracted for further analysis and the GI list was transformed into gene ontology using the home-application TBtools (http://cj-chen.github.io/TBtools/). Hammer was also applied to search a conservative domain collected in a pfam database of all unigenes.

### Gene expression analysis and detection of co-expresssion network

Differential expression analysis of two tissues was performed using the DESeq (Anders and Huber, 2010). A corrected *P*-value of 0.05 and abs [log_2_ (Fold change)] of 1 were set as the threshold for significantly differential expression. All differentially expressing genes were clustered based on their FPKM values with the k-means method. Log2-tranformed the FPKM of genes that were differentially expressing in at least one pairwise comparison in 11 tissues as per Langfelder and Horvath (2008). All parameters were set as defined except “soft_power = 15, deep_split = 3, min_module_size = 30, ME_miss_thread = 0.2.”

### Quantitative real-time PCR (qRT-PCR) analysis

A SYBR Primix Ex Taq kit (Takara), in accordance with manufacturer instructions, was used to do quantitative real-time RT-PCR. Gene-specific primer pairs were designed for specific unigenes and *actin* (Supplementary Table 1). Amplification was carried out with the following cycling parameters: denaturing for 3 min at 95°C, 40 cycles of denaturation at 95°C for 15 s, annealing for 20 s at 58°C, and extension at 72°C for 30 s. The molecular masses of the products were confirmed using the electrophoresis method. The melting curves of the products were also analyzed. To ensure reproducibility of results, quantitative PCR experiments were performed in triplicate for each sample, and the expression values obtained were normalized against *actin*. To assess the relative gene expression, a 2^−ΔΔ^Ct method was used (Livak and Schmittgen, 2001). Tissues used for RT-qPCR were the same batch of plants as used for RNA-seq.

## Results

### RNA-sequencing and *de novo* assembly of chinese kale transcriptome for the whole plant and individual tissues

The plants of Chinese kale (*B. oleracea* var. *alboglabra* Bailey cv. Zhonghua) were sampled at the commercial stage when the plants were tender and fully grown with inflorescence as high as the apical leaves (Yin et al., 2015). Since different plant parts, including those from the same organ, differ significantly in glucosinlates, and other nutrient quantity (Sun et al., 2011a, 2012), plants were divided into 11 parts (hereafter named as tissues, Supplementary Image 1) for transcriptome analysis: senescent leaves (SL), mature leaves (ML), young leaves (YL), leaf veins(LV), petioles (Pe), young bolting stem flesh (YB), middle bolting stem flesh (MB), bolting stem skin (BS), flower buds (FB), combining sites (CS), and roots (Ro). The transcriptomes of all tissues were subjected to sequencing and digital gene expression profiling. High-quality 150-bp clean reads from 17.8 to 21.5 million, with an average of 19.6 million, were obtained for each of the 11 tissues (Supplementary Table 2). The raw data was submitted to NCBI-SRA database with the BioProject accession number PRJNA358667. Each of the transcriptomes contains 47.62~48.96% GC contents, with an average GC content of 48.36%. The Q30 of the 11 samples were 95.59~96.09% with an average of 95.77%, which indicated that the quality of the RNA-sequencing was adequate for *de novo* assembly and expression analysis. A total of 216 million 150-bp clean reads were obtained from the 11 tissues (Supplementary Table 2), representing 65 Gb sequences that are equivalent to >102.5x of the *B. oleracea* 0.63-Gb genome (Liu et al., 2014) and a total of 17,794,601~21,522,897 clean reads were obtained for each group of tissues with an average of 19,655,227 clean reads. This coverage of RNA sequencing was sufficient for accurate gene expression profiling and *de novo* assembly. Because the entire genome sequence of Chinese kale was unknown, all clean reads were *de novo* assembled with the Trinity method (Grabherr et al., 2011). From the clean reads, 49,582~98,423 unigenes were assembled for each tissue with an N50 of 915~1211 bp and an average length of 562~770 bp and 98,180 unigenes were assembled for the whole plant from the unigenes of all 11 tissues, with an N50 of 1240 bp and an average length of 820 bp (Supplementary Table 3). These results indicated that the N50 and average length of unigene from whole plants were much longer than those of any specific tissue. The 98,180 unigenes of entire plants obtained in the present study were used as a reference sequence to align and identify the sequencing reads. This allowed for the mapping of 92.89~98.66% of the clean reads that passed through our filters and mapped to reference sequences, representing 32,509,572 and 40,510,710 reads for 11 tissues, respectively (Supplementary Table 3).

### Functional annotation of the unigene sets derived from the whole plant

To understand the global expression patterns of the Chinese kale transcriptome in a view of systems biology, functional annotation of the assembled unigenes were submitted against public databases. The results showed that a total of 80,688 (82.18%) unigenes were significantly similar to a sequence in at least one of the public protein databases, including Nr (NCBI non-redundant protein), GO (Gene Ontology), COGs (Clusters of Orthologous Groups), Swiss-Prot protein, and the KEGG (Kyoto Encyclopedia of Genes and Genomes; Supplementary Table 4, Supplementary Image 2). The annotation results showed that 78,900 (80.36%) unigenes were found to have significant similarity with protein sequences in the Nr database at a cutoff of *E* ≤ 1e-05 and 58,063 (59.14%) unigenes were annotated by swissprot database (Supplementary Table 4, Supplementary Image 2). Most of the genes were annotated by more than one database. For example, 11,845 unigenes were annotated by swissprot and NR database, 10,037 unigenes by swissprot, NR, and KEGG database, only 15375, 719, and 424 genes were specifically annotated by Nr, swissprot, and KEGG respectively, and no unigene was specifically annotated by the GO or COG database, which indicated that unigenes were much more easily annotated by NR and swissprot than other databases. The majority of the annotated sequences corresponded to the known nucleotide sequences of plant species, with 51.73, 27.84, and 5.79% matching with *B. napus, B. oleracea*, and *B. rapa* respectively (Supplementary Image 2B). The top three Blast hits were *Brassica* species, suggesting that the transcript sequences of Chinese kale obtained in the present study were correctly assembled and annotated.

A home-made application was used to obtain GO terms for all assembled unigenes from whole plants and a total of 30,272 (30.83%) unigenes were assigned one or more GO terms. In the present study, all the GO terms were classified into 49 functional groups including biological processes (19 sub-groups), cellular components (17 sub-groups), and molecular functions (13 sub-groups; Supplementary Image 3), with multiple terms assigned to the same unigenes. Within the biological processes classification, the most abundant groups of transcript sequences were metabolic (20,312) and cellular processes (17,798). Among the category of molecular function, the largest number of GO terms were related to binding (16,581) and catalytic activity (14,237). For the cellular components, the majority of assignments were mostly assigned to cell (11,978) and cell part (11,790). These results suggested that the majority of GO classifications involved in the annotated unigenes were taking responsibility for basic biological regulation and metabolism.

The COG database is built on coded proteins with complete genomes as well as system evolution relationships of bacteria, algae, and eukaryotes (Dutkowski and Tiuryn, 2007). Overall, 18,728 (25.14%) unigenes were annotated by the COG database (Supplementary Table 4, Supplementary Data Sheet 1). A total of 30,112 functional annotations were produced in 25 COG categories. Most of these unigenes were annotated by several COG functions. Among these COG categories, the largest group is the cluster for “General functions prediction only” associated with basic physiological and metabolic functions, followed by “Signal transduction mechanism,” “Post-translational modification, protein turnover, chaperones,” “Translation, ribosomal structure and biogenesis,” and “Carbohydrate transport and metabolism.” Very few unigenes were assigned to the “RNA processing and modification” and “Chromatin structure and dynamics” categories (Supplementary Image 5).

KEGG annotated analysis were conducted for all tissues and KEGG pathway mapping assigned 18,516~27,362 of the 49,582~8423 unigenes of different tissues with K number assignments to 3705~4551 K num involved in 138 metabolic pathways. Of those pathways, ko00966 (Glucosinolate biosynthesis) is likely to significantly contribute to glucosinolate biosynthesis. Pe, Ro, and BS have the largest number of unigenes, with 169 (0.62%), 147 (0.54%), and 143 (0.53%), respectively assigned to pathway ko00966 (Table 1). YB contained the smallest number of unigenes, with 88 (0.38%) and 98 (0.41%) of the total annotated genes (Table 1). These results indicated that glucosinolate biosynthesis genes in Chinese kale might be most active in Pe, Ro, and BS tissues. The pathway ko00942 (Anthocyanin biosynthesis) is ostensibly involved in anthocyanin biosynthesis, with the leaves (YL, ML, and SL) containing more of these genes than the roots and bolting stem (YB and MB). The pathway ko00941 is likely to contribute significantly to flavonoid biosynthesis. CS, Ro, and SL tissues have more ko00941 genes annotated than in YL, ML, YB, and MB, which indicated that flavonoid may be mainly synthesized in older tissues of Chinese kale.

**Table 1:** **Statistics of pathway mapping of the unigene sets derived from different tissues**.

**Sample name**	**No. of Unigenes having K num**	**No. of K num**	**KEGG pathways**	**ko00966 (Glucosinolate biosynthesis)**	**ko00942 (Anthocyanin biosynthesis)**	**ko00941 (Flavonoid biosynthesis)**
FB	26,288	4551	138	117 (0.45%)	43 (0.16%)	230 (0.78%)
YL	24,352	4349	138	100 (0.41%)	46 (0.19%)	163 (0.67%)
ML	18,516	3705	138	86 (0.46%)	31 (0.17%)	123 (0.66%)
SL	24,806	4541	138	120 (0.48%)	44 (0.18%)	196 (0.79%)
LV	25,430	4525	138	137 (0.54%)	46 (0.18%)	194 (0.76%)
Pe	27,285	4571	138	169 (0.62%)	45 (0.16%)	219 (0.80%)
YB	23,667	4307	138	98 (0.41%)	33 (0.14%)	153 (0.65%)
MB	22,993	4291	138	88 (0.38%)	26 (0.11%)	156 (0.68%)
BS	27081	4430	138	143 (0.53%)	40 (0.15%)	201 (0.74%)
CS	24,107	4393	138	108 (0.45%)	37 (0.15%)	200 (0.83%)
Ro	27,362	4774	138	147 (0.54%)	33 (0.12%)	231 (0.84%)

### Expression profiles of the unigenes in different tissues

**Figure 1:**
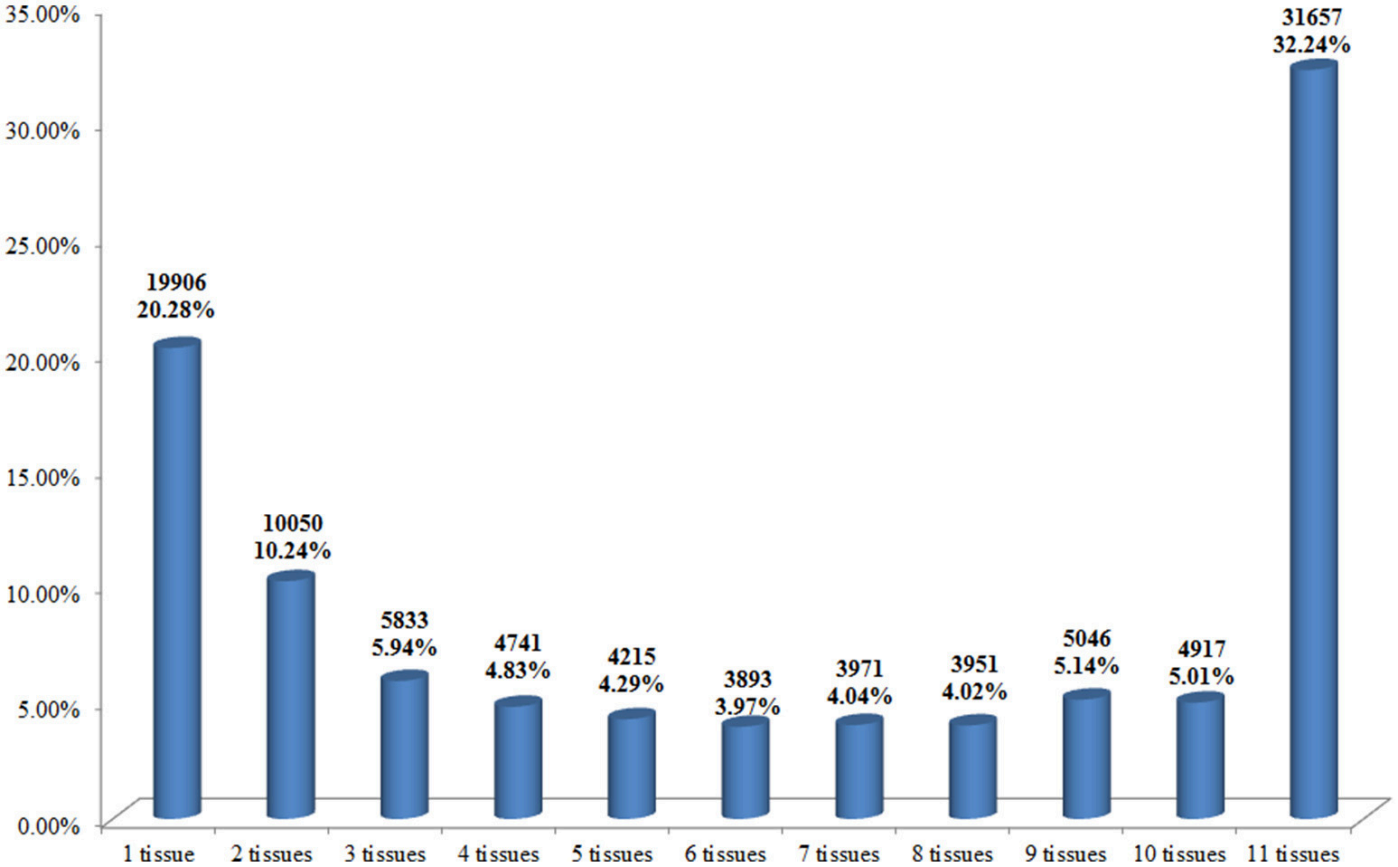
**Number and percentage of unigenes specifically expressed in 11 different tissues**.

The expressions patterns of the 98,180 unigenes in different tissues were digitally measured and their expression relationships were characterized. The expression levels of most unigenes varied by several fold among different tissues (Supplementary Data Sheet 1). A total of 19,906 unigenes were shown to be specifically expressed in one of the 11 tissues, representing 20.28% of the 98,180 unigenes (Figure 1). Of the 98,180 unigenes, 31,657 (32.24%) expressed in all 1l tissues studied, indicating that most of these genes in Chinese kale were basically expressed in all of the tissues (Figure 1). A total of 46,617 unigenes were identified to be expressed in 2–10 of the 11 tissues studied, together accounting for 47.48% of the 98,180 unigenes (Figure 1). Of the one tissue specifically expressing 19,906 unigenes, the numbers of unigenes varied by more than 37 times, from 406 in ML to 4498 in FB tissues (Supplementary Table 5). It was also observed that the number of unigenes specifically expressed in flower buds and roots was much larger than that specifically expressed in other tissues studied (Supplementary Table 5). The tissues Pe and BS shared the largest number of genes (2264); Ro and CS had 873 genes in common, while ML and YB shared only 17 specifically expressed genes.

**Figure 2:**
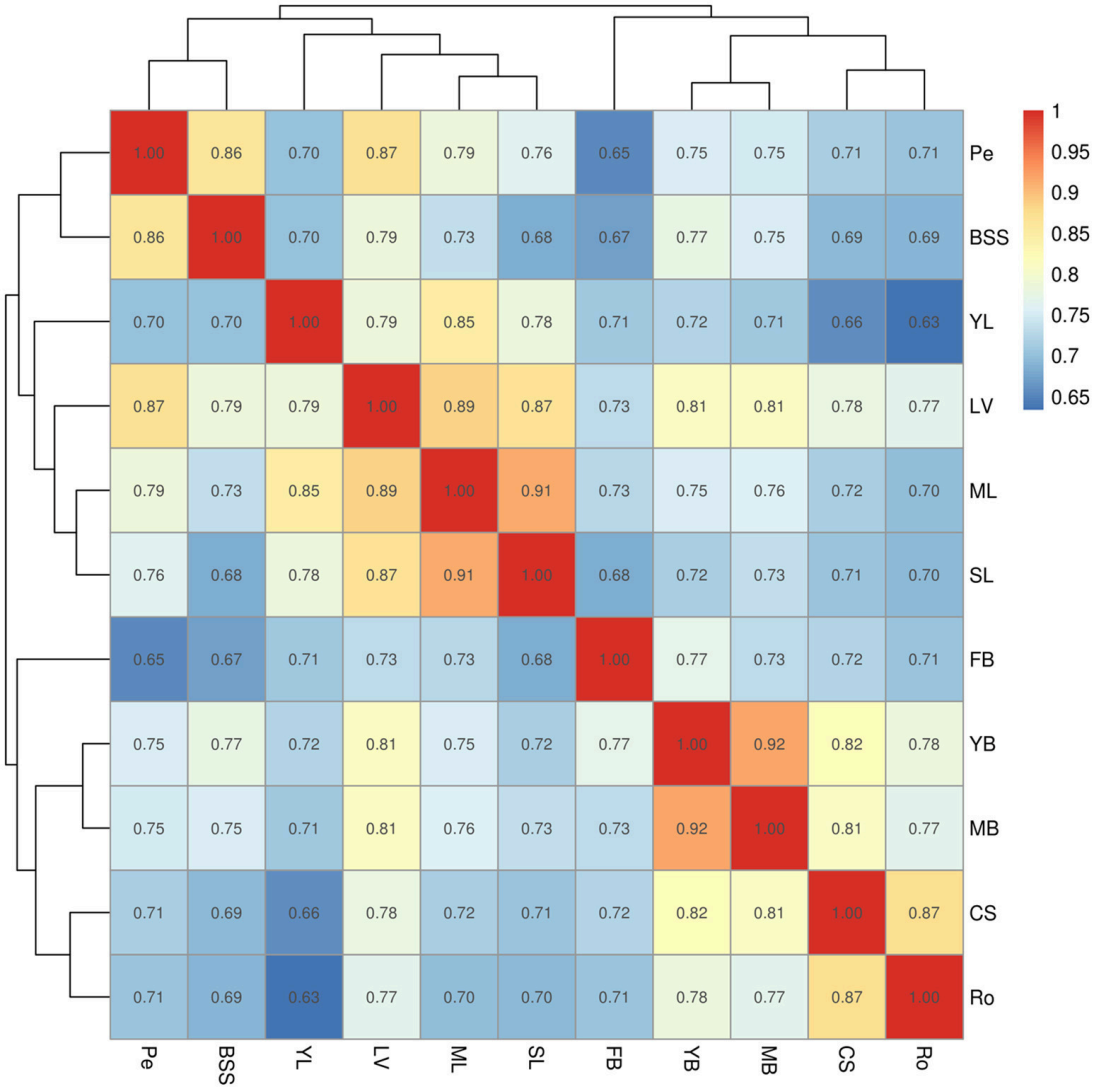
**Hierarchical clustering on pairwise correlation coefficients of unigene expression data from Chinese kale samples**. Numbers in the cells represented the spearman's rank correlation coefficient. Sample identifiers: SL, senescent leaf; ML, mature leaf; YL, young leaf; LV, leaf vein; Pe, petiole; YB, young bolting stem flesh; MB, middle bolting stem flesh; BS, bolting stem skin; FB, flower budsl; CS, combining sites; Ro, Roots.

Relationships of unigene expression among tissues were determined by calculating correlation coefficients of gene co-expression, using hierarchical clustering on pairwise correlation coefficients of unigene expression data from samples (Figure 2). The relationships between samples were displayed by numbers at nodes representing approximately unbiased *P*-values obtained by multi-scale bootstrap resampling. The 11 samples were divided into two main groups: group I (YL, SL, LV, ML, Pe, and BSS) and group II (FB, YB, MB, CS, and Ro tissues). Group I was further divided into two groups: YL, SL, LV, and ML were clustered in one clade, and Pe, BSS were clustered in another clade. As expected, ML and SL were highly similar. However, YL showed a closer relationship with LV than ML and SL, indicating the biology process in YL were different from ML and SL. As expected, YB and MB were highly similar. FB was the most unique tissue in Chinese kale.

### Clustering of unigenes by expression level illustrated metabolic pathways in different tissues

**Figure 3:**
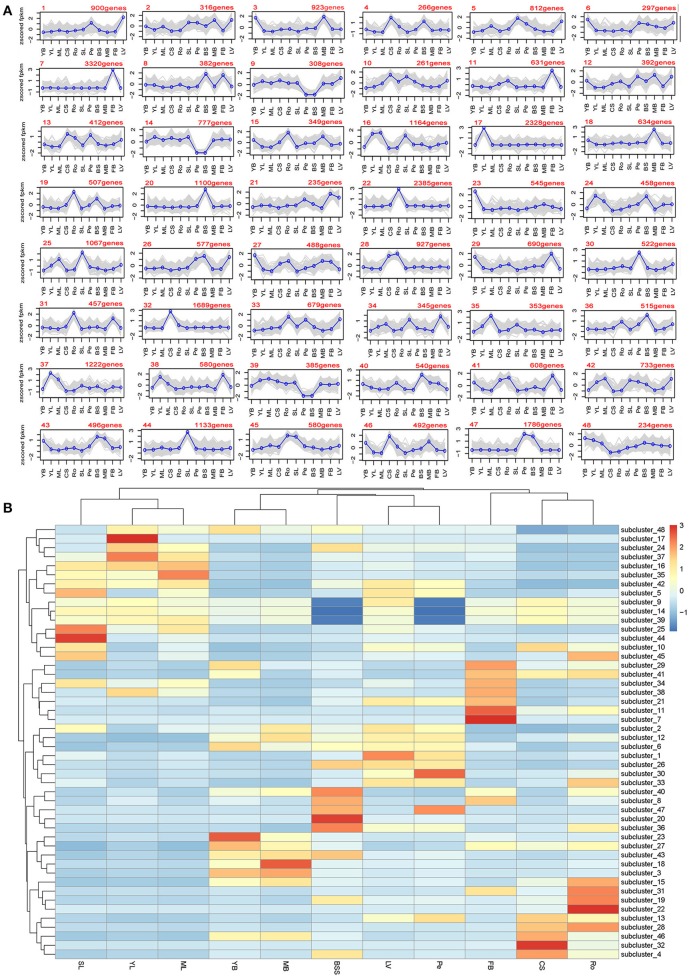
**Clustering analysis of gene expression profiles. (A)** Cluster analysis of differentially expressed genes with the K-means method; **(B)** Heat map illustrating the expression profiles of the genes in each cluster.

A set of genes with similar expression levels are often functionally correlated. Novel candidate genes whose functions correlate with the development of different tissues were selected. All 98,180 unigenes were clustered using the software MultiExperiment Viewer. The K-means method and hierarchical clustering were employed. A total of 48 clusters were obtained from the expression data of the 11 tissues (Figure 3A). Overall, there were distinct gene expression peaks in different tissues. For example, flower bud tissues have the highest cluster number with expression peaks. This tissue might have the most complex and special biology process; leaf veins and middle leaves have only one cluster with expression peaks (Figure 3A). A heat map was created to illustrate the variations of gene expression in each tissue (Figure 3B). As expected, YL, ML, and SL were clustered together with similar variations of gene expression, while YB and MB, CS and Ro were clustered together, respectively. These results corresponded to the results of unigene expression data (Figure 2) and to the rules of plant growth and development. For example all leaves (YL, ML, and SL) clustered together and bolting stems (YB and MB) clustered together, which indicated that the expression data obtained were accurate.

### Co-expression network analysis of unigenes in different tissues with WGCNA

**Figure 4:**
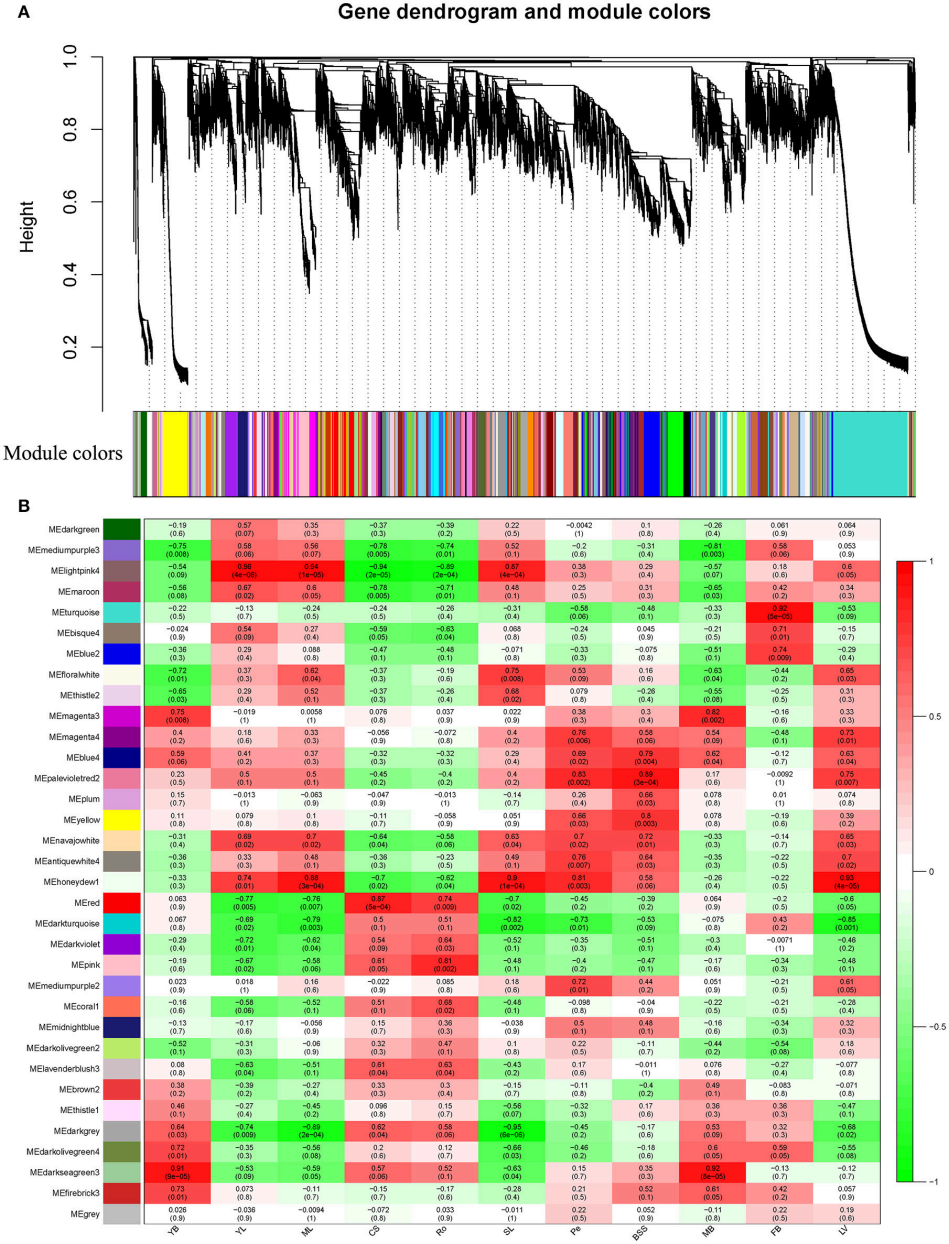
**Detection of co-expression network in whole plant of Chinese kale. (A)** Hierarchical cluster tree showing co-expression modules identified by WGCNA analysis. Each leaf in the tree is one gene. The major tree branches constitute 34 modules labeled by different colors. **(B)** Module-tissue association. Each row corresponds to a module. Each column corresponds to a specific tissue. The color of each cell at the row-column intersection indicates the correlation coefficient between the module and the tissue type. A high degree of correlation between a specific module and the tissue type is indicated by dark red or dark green.

WGCNA (Langfelder and Horvath, 2008), a systems biology approach, was used to analyze the relationships and networks between different genes. In the present study, co-expression networks were built on the basis of pairwise correlations among genes according to the trends of gene expression in all examined tissues. Genes with high correlation coefficients that indicated a high degree of interconnections between the genes were defined as modules. As seen in the dendogram on Figure 4A, 34 unique modules were identified in the analysis, with each module depicted by a branch of different color and each gene depicted by a leaf. Each module's gene expression profile is represented by its eigengene, its most notable component. The 34 resulting eigengenes each correlate with unique tissue types due to their tissue-specific expression profiles (Figure 4B). Notably, 5 co-expression modules are comprised of genes that are highly expressed in a single tissue type, including MEturquoise in FB, MEhoneydew1 in MB, MEpink in Ro, MEred in CS, MEyellow in BS, and 4 co-expression modules are comprised of genes that are highly expressed in 2~3 tissue types (*r* > 0.8; Figure 4B). Therefore, each of these 9 modules identifies a specific tissue or a cluster of genes in 2~3 similar tissues. For example, 1383 genes involved in the MEturquoise module were highly specifically accumulated in flower buds, which indicated that this group of genes might be responsible for flower buds or anther development. A total of 1331 genes involved in the MEdarkseagrean3 module were highly specifically accumulated in young bolting stems and middle bolting stems, which indicated that these groups of genes might be responsible for bolting stems development. A total of 2968 genes involved in MElightpink module were highly specifically accumulated in young, middle and senescent leaves, which indicated that this group of genes might be involved in the aging process in leaves (Figure 4B). These results were consistent with the K-means clustering analysis, in which all leaves (YL, ML, and SL) and bolting stems (YB and MB) clustered together, respectively.

### Analysis of the genes likely involved in glucosinolate metabolism and regulation

**Figure 5:**
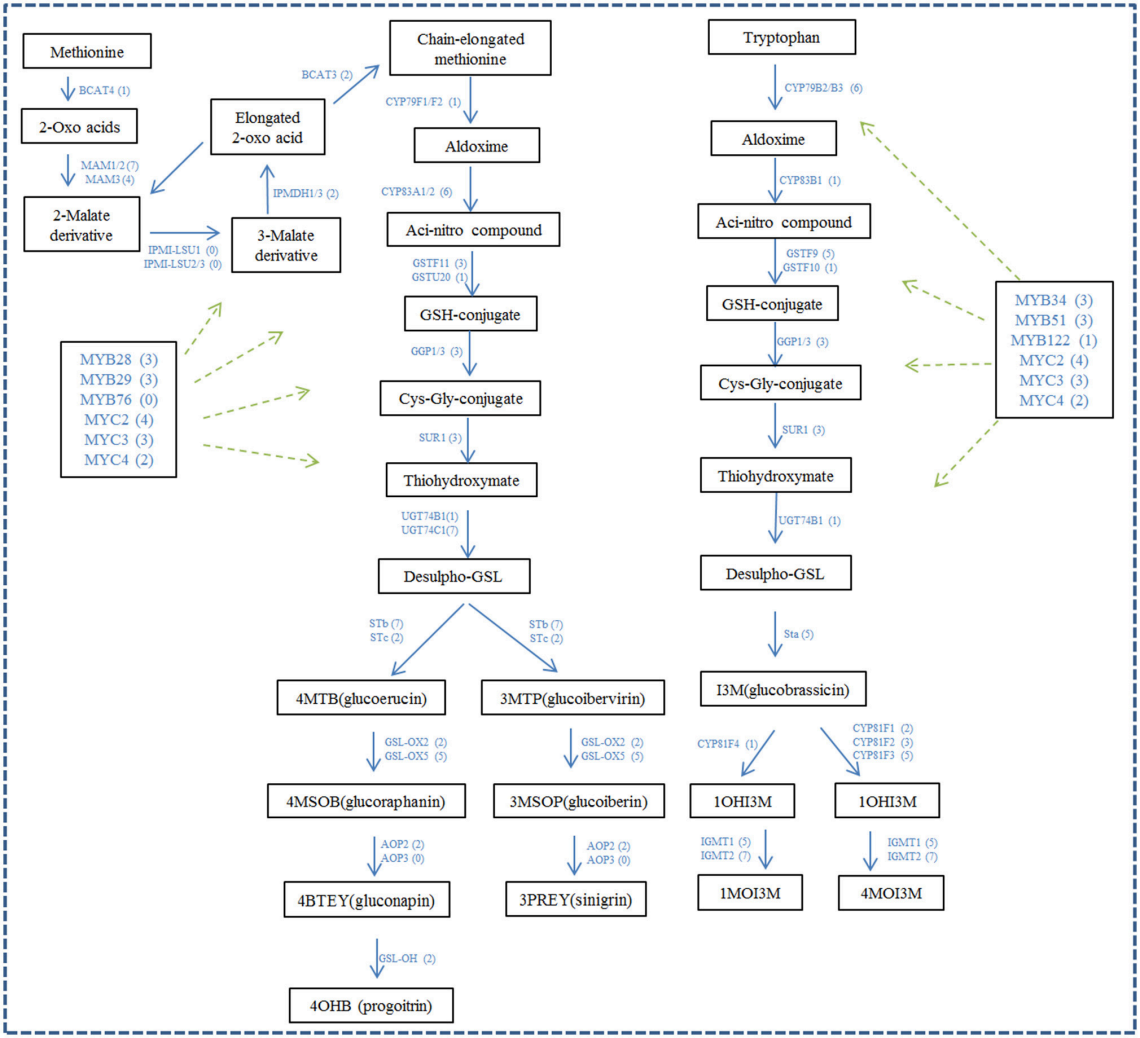
**The unigenes likely involved in glucosinolates biosynthesis and its regulation**. The figure is adapted from Sønderby et al. (2010), Yatusevich et al. (2010), Schweizer et al. (2013), and Liu et al. (2014). The numbers in brackets following each gene name indicate the number of unigenes annotated to that gene.

A total of 106 unigenes in our dataset were identified to be homologous with the 42 genes encoding glucosinolate biosynthesis genes in *A. thaliana*. Twenty-seven unigenes were identified to be homologous with the 6 genes involved in glucosinolate biosynthetic Co-substrate pathways of *Arabidopsis* (Figure 5 and Supplementary Data Sheet 2). The genes related to glucosinolate metabolism in *A. thaliana* have 0~7 homologous unigene in Chinese kale with an average ratio of about 3 copies of unigenes in Chinese kale for one in Arabidopsis. For example, *MAM1/2, STb*, and *IGMT2* each have 7 homologous unigenes in Chinese kale, while IPMDH3, CYP83B1, and GSTF10 have only one and LeuD2, FMOGS-OX2, FMOGS-OX3, and FMOGS-OX4 have no homologous unigene in Chinese kale. Furthermore, 46 unigenes were identified to be homologous to the 13 genes encoding myrosinase (Figure 5 and Supplementary Data Sheet 2). The myrosinase genes in *A. thaliana* have 1~7 homologous unigene in Chinese kale with an average of about 3 unigenes. In addition, the transcript factor regulating glucosinolate biosynthesis was identified from the dataset of Chinese kale. A total of 32 unigenes were identified to have homology with 12 transcript factors from Arabidopsis, with an average of 2.6 unigenes for one transcript factor. Notably, except *MYB76*, every transcript factor has 1~4 unigenes annotated in Chinese kale (Figure 5 and Supplementary Data Sheet 2).

**Figure 6:**
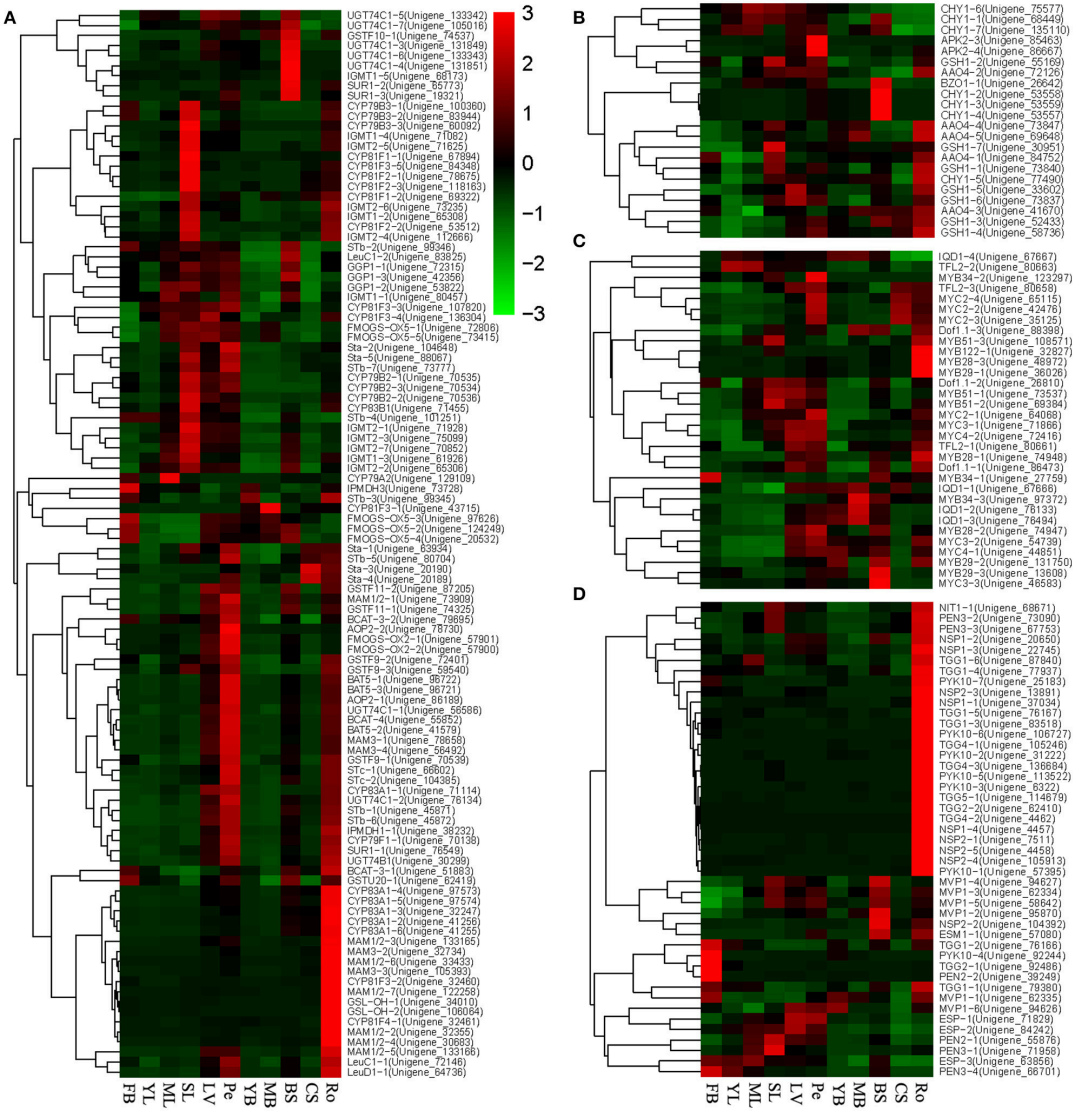
**Heatmaps of genes likely involved in glucosinolate regulation and metabolism constructed based on their expressions in 11 tissues. (A)** Genes likely involved in three main steps of glucosinolate biosynthesis; **(B)** Genes likely involved in GS biosynthetic Co-substrate pathways; **(C)** Transcription factors likely involved in regulation glucosinolate biosynthesis; **(D)** Genes likely involved in GS breakdown.

The expression data of the 136 genes involved in glucosinolate biosynthesis and 46 unigenes involved in glucosinolate breakdown in 11 tissues were selected. The expression relationships and levels of these genes were examined using the heatmap method (Figure 6). For the glucosinolate biosynthesis genes, the expressions of the genes varied by multiple fold among different tissues. Interestingly, for the glucosinolate biosynthesis, a large number of genes, including *CYP83A1, MAM1/2/3, GSL-OH, CYP81F3/4*, and so forth, have very high expression level in the root. Many genes, including *CYP79B2/3, IGMT1/2, CYP81F1/2/3, FMOGS-OX5, AAO4-2, GSTF9/10, Sta*, and so on, were highly expressed in SL, and a set of genes, including *APK1/2, CYP83A1, UGT74B/C1, STb, BCAT3/4, AOP2/3, GSTF9/11, STc, FMOGS-OX1, GSH1, LeuC/D1, IPMDH1, CYP79F1, SUR1*, and so on, were highly expressed in Pe. However, only a few glucosinolate biosynthesis unigenes were highly expressed in other tissues, including leaves and bolting stems (Figure 6A), which indicated that the glucosinolate biosynthesis occurs more strongly in Ro, Pe, and SL than other tissues. Twenty-six (56.52% of the GS breakdown genes) unigenes were clustered in a big clade with expression peaks in root, such as *NIT1, PEN3, PYK10, NSP1, NSP2, TGG1, TGG2, TGG4, TGG5*, and so on (Figure 6D). Six unigenes, *MVP1, NSP2*, and *ESM1* were clustered together in another clade, with an expression peak in BS (Figure 6D). Four unigenes, *TGG1, PYK10, TGG2*, and *PEN2*, were highly expressed in FB, which suggested that similarly to glucosinolate biosynthesis, the breakdown of glucosinolate might happen more frequently in the root than in other tissues. The expression data of all the unigenes annotated by transcript factors for gulcosinolate biosynthesis were also extracted and a heatmap was built. Unlike the genes involved in GS biosynthesis and breakdown, the clustering of these unigenes was complex and disordered. Most of the unigenes annotated by the same transcript factors had similar expression patterns, such as *MYC2, IQD1, MYB29, MYB51*, and so forth (Figure 6B). However, some unigenes were annotated to the same transcript factors with different expression patterns, such as *MYB34, MYB28*, and *Dof1.1*. Interestingly, there were 3 unigenes annotated by *MYC*2, 3, and 4, respectively, with similar expression patterns, which indicated that these 3 unigenes were the direct homologous of *MYC2, 3*, and *4*, and might play similar roles with *MYC2, 3*, and *4* in *Arabidopsis*.

### Validating the gene expression patterns by qRT-PCR

**Figure 7:**
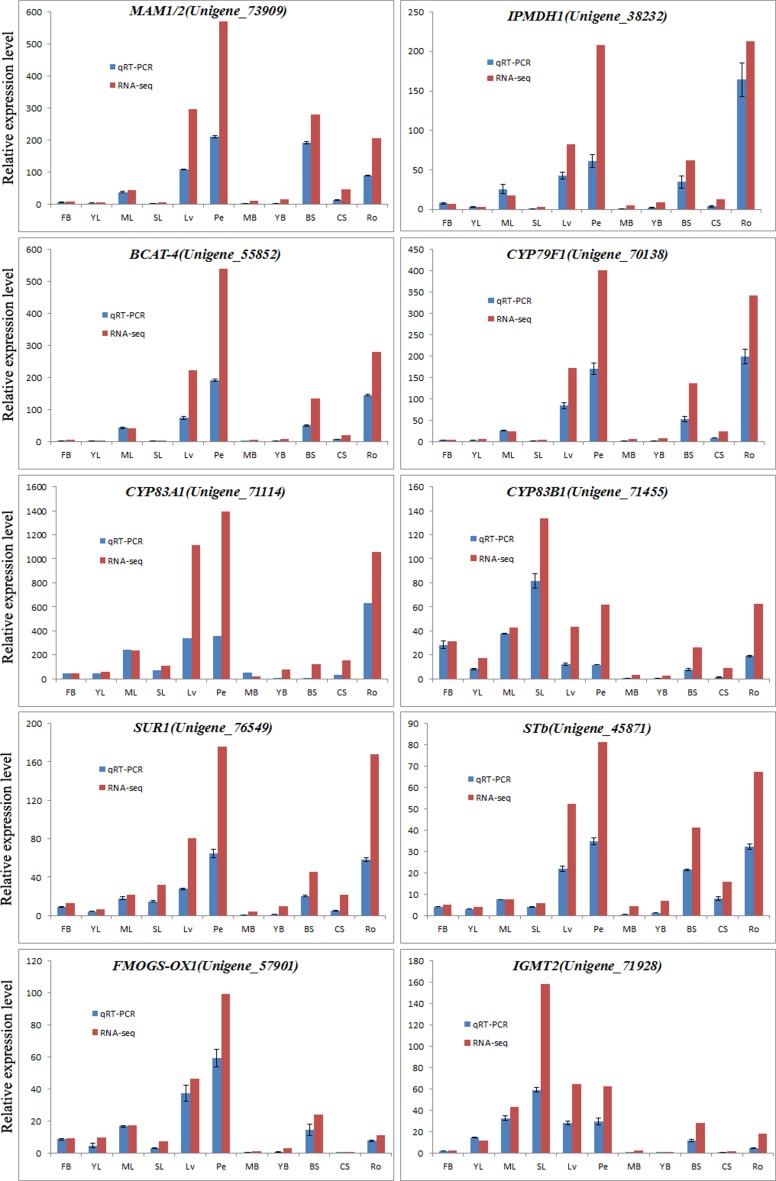
**Expression analysis of glucosinolate biosynthesis-related genes determined by qPCR and RNA-seq**. These 10 genes were random selected from the genes annotated by the glucosinolate biosynthetic genes in Arabidopsis (Supplementary Data Sheet 2). The blue and red bars were representing the unigene expression patterns in 11 tissues by qPCR and RNA-seq, respectively.

To further validate the expression patterns of the glucosinolate biosynthetic genes revealed by RNA sequencing, qRT-PCR was conducted to examine the expression levels of 10 genes in 11 tissues of Chinese kale. All these 10 genes, Unigene_73909 (*MAM1/2*), Unigene_38232 (*IPMDH1*), Unigene_55852 (*BCAT-4*), Unigene_70138 (*CYP79F1*), Unigene_71114 (*CYP83A1*), Unigene_71455 (*CYP83B1*), Unigene_76549 (*SUR1*), Unigene_45871 (*STb*), Unigene_57901 (*FMOGS-OX1*), and Unigene_71928 (*IGMT2*), were randomly singled out from the genes annotated by the glucosinolate biosynthetic genes (Supplementary Data Sheet 2). As shown in Figure 6, the results of qRT-PCR were well in accordance with the expression data obtained by RNA-Seq. The expression of all these 10 genes in Chinese kale exhibited similar patterns among different tissues with the highest level of expression in Ro, Pe, or LV (Figure 7), and this is because all of these genes might be involved in the same biological event of glucosinolate biosynthesis.

## Discussion

### Building a gene data resource and annotation

The Chinese kale was thought to belong to *B. oleracea*, and the genomic sequence of *B. oleracea* sp. Capitata were obtained recently (Liu et al., 2014). However, the botanical characteristics of Chinese kale are notably different, indicating differences in gene expression patterns. Unprecedentedly, we have sequenced the genes expressed in the Chinese kale plant, and a total of 216 million clean reads were obtained from the 11 distinct tissues, representing 65 Gb sequences. This is equivalent to >102.5x of the *B. oleracea* 0.63-Gb genome (Liu et al., 2014; Supplementary Table 2). This coverage of RNA sequencing proved to be sufficient for accurate gene expression profiling and *de novo* assembly in the previous study (Wang K. et al., 2015). A total of 98,180 unigenes were obtained from the plants. The number of transcripts exceeded the results (73,084 unigenes) of the Wang et al. (2013) radish (*Raphanus sativus* L.) study and was smaller than the 248,993 unigenes generated from 14 tissues of ginseng (Wang K. et al., 2015), which indicated we had well-assembled the unigenes from each of the 11 tissues of Chinese kale. The average unigene size (820 bp) was longer than those in *Cymbopogon winterianus* (714 bp; Devi et al., 2016) and *Pueraria lobata* (683) (Wang X. et al., 2015), which indicated that the quality of the assembled results was high.

More than 80% of the unigenes were annotated by known proteins from Nr, swissprot, GO, and other databases (Supplementary Image 5), and most of these genes were annotated by *Brassica* plants, which indicated the sequencing and assemble results were reliable. The rate of annotated unigenes in Chinese kale was lower than the range of the assembled transcript sequences in radish (92.09%) (Wang et al., 2013) and *C. winterianus* (92.05%) (Devi et al., 2016) but higher than in other non-model species, such as 58% in safflower flowers (Li et al., 2012), 58.01% in Chinese fir (Huang et al., 2012), and 56.51% in spinach (Yan et al., 2016), suggesting the relatively conserved functions of the assembled unigenes in Chinese kale. The unigene without any hits to known proteins may represent new genes or genes with assembly problems (Wang et al., 2013). Genomic resources and tools are essential for many aspects of advanced research of an organism. Nevertheless, to date, only 30,759 ESTs for Chinese kale (*B. oleracea* var. *alboglabra*) have been uploaded to the NCBI database. This study provides not only a comprehensive set of 98,180 unigenes for the entire Chinese kale plant, in addition to the unigene sets of 11 different tissues, but also the expression profiles of every gene of the 98,180 unigene sets in the 11 tissues. These results will significantly enhance the gene database of Chinese kale currently available in GenBank and to the public, and will provide a comprehensive gene expression database.

### The global expression patterns for all the unigenes in different tissues

Our study is the first account of gene expression patterns in various Chinese kale tissues sampled at the stage of commercial harvest. The data indicated that despite the drastic variability in the number of gene expressed in various tissues (Supplementary Data Sheet 1), there was also a remarkable consistency in the percentage of genes categorized into each GO term of the functional category regardless of the tissue origin (Supplementary Images 3, 4). These results were in agreement with the previous study in Ginseng (Wang K. et al., 2015), which determined that appropriate and consistent assignment of genes to biological processes appear to be a key factor in supporting the regular functioning of a cell (Wang K. et al., 2015). As seen via gene tissue-specific expression analysis, more than 67% of the genes in Chinese kale are specific to one or more tissues (Supplementary Image 5, Supplementary Table 5), indicating that different gene clusters in different tissues are responsible for different functional biology processes. This was especially true in root and flower buds, which have the largest number of tissue-specific genes. This finding likely indicates that these two organs have more specialized biological processes than other organs. As expected, a significant percentage of the genes (32% of the total) were expressed consistently in different tissues. These genes are considered to be house-keeping genes, and their number was fewer than in Ginseng studies (nearly 60% of the genes, Wang K. et al., 2015). Genes of the Chinese kale cells with correlated actions de facto form co-expression networks, which can be further categorized into even more specific interaction clusters using functionality criteria (Figures 2, 6). Although, the functions of these unigenes are still unknown, the clustering results may provide information useful for isolation of genes contributing to the same trait or biological process. The clustering of different tissues by global gene expression data indicated that all 11 tissues were divided into 2 groups and that leaves (YL, ML, and SL) or bolting stems (YB and MB) were clustered together. These results were conformed both by calculation of correlation coefficients and the WGCNA analysis (Figures 3, 7), which suggested that the results of gene expression were reliable and correct. Interestingly, the gene expression model in FB was largely different from the model in other tissues, which indicated that the biological processes in FB were complex and specialized.

### Analysis of the genes involved in glucosinolate metabolism

In recent years, the main pathway of glucosinolate biosynthesis has been extensively studied in *A. thaliana*, and many critical genes have been successfully identified and functionally characterized (Sønderby et al., 2010). Based on the genes in *Arabidopsis*, many homologous genes involved in GS metabolism were identified in *B. rapa* (Wang H. et al., 2011), Cabbage (*B. oleracea* var. capitata, Liu et al., 2014), and *R. sativus* (Wang et al., 2013), as a result of the sequencing technology developed in recent years. The biosynthesis of glucosinolate occurs via three independent stages: (i) chain elongation of precursor amino acids Met and Phe, (ii) formation of the core glucosinolate structure, and (iii) secondary modifications of the amino acid side chain (Sønderby et al., 2010). In the present study, a total of 181 unigenes likely involved in glucosinolate metabolism were isolated from the Chinese kale sequence data. There is an average of three Chinese kale unigenes for one gene in Arabidopsis. Generally, multiple unigenes were annotated by the same gene and these unigenes may represent different fragments of a single transcript or different members of a gene family (Hyun et al., 2012; Wang et al., 2013). Based on the conserved syntenic block analysis between the genomes of *Brassica* plants and *A. thaliana*, the hypothesis of an ancestral karyotype was proposed and comparative physical mapping studies confirmed genome triplication in *B. rapa* from *Arabidopsis* (Park et al., 2005; Schranz et al., 2006). Multiple copies of the glucosinolate genes of *B. rapa* may have resulted from genome triplication, and retained synteny with their orthologs in *A. thaliana*, whereas most genes that appeared to contain fewer than three copies of genes might be caused by gene loss following triplication (Wang H. et al., 2011). This hypothesis was further proved by the present study, in which there was found to be an average of three glucosinolate metabolism related unigenes in Chinese kale for one ortholog in *Arabidopsis*. In the present study, most of the genes involved in GS biosynthesis and breakdown in Chinese kale were identical in *Arabidopsis, B. rapa*, and Cabbage (Liu et al., 2014), and some glucosinolate genes (MYB76, FMOGS-OX1, FMOGS-OX3, and AT1G62570.1, IPMI LSU1, IPMI SSU2, IPMI SSU3) had no homologous unigene found in Chinese kale RNA-seq data. These genes were also not present in the genome sequence of *B. rapa* (Wang H. et al., 2011) and Cabbage (Liu et al., 2014), which indicated that some genes might be lost during the evolution of brassica plants from *Arabidopsis*. Although, the number of genes in one gene family was different in *B. rapa*, Cabbage, and Chinese kale (Supplementary Data Sheet 2), our results were still in accordance with the results from *B. rapa* (Wang H. et al., 2011) and Cabbage (Liu et al., 2014). Interestingly, we found that five Chinese kale unigenes (Unigene_84752, Unigene_72126, Unigene_41670, Unigene_73847, and Unigene_69648) annotated by *Arabidopsis* glucosinolate biosynthesis genes, *AAO4*, were present in *B. rapa* but not in Cabbage (Liu et al., 2014), which indicated that there may be a difference in glucosinolate biosynthesis between different Brassica plants. The pathway of glucosinolate biosynthesis was very well-conserved in the Brassicaceae family but also distinct in certain *Brassica* species because of the difference in the secondary modifications of the amino acid side chain (Sønderby et al., 2010).

The genes regulating glucosinolate metabolism were for the first time characterized by the present study in expression and expression correlations in different tissues of Chinese kale. Although, further studies will be needed to confirm the functions of these genes, the genes identified from the gene sequences will provide candidates for isolation of the genes involved in glucosinolate metabolism. The present study has shown that most of the glucosinolate biosynthesis genes, such as *CYP83A1, MAM1/2*, and *GSL-OH*, and the breakdown genes, such as *PKY10, TGG1/2/4/5, NSP1/2, PEN3*, and so forth (Figures 6A,C), had high expression level in the root. These results were in agreement with earlier findings that roots of Chinese kale contained markedly higher concentrations of glucosinolates, especially gluconasturtiin and glucoerucin, than other organs (Sun et al., 2011a). These results indicated that biosynthesis and breakdown processes happen more intensively in root than in other tissues of Chinese kale. However, we did not see a notably higher expression level of the transcription factors involved in glucosinolate biosynthesis in the root (Figure 6B). We therefore suggested that the transcription factors might regulate the GS metabolism genes at a relatively low level.

Generally, one *Arabidopsis* glucosinolate gene had multiple (3 or more) homologous genes in Chinese kale (Supplementary Data Sheet 2 and Figure 5), and most of the homologous genes had a similar expression model and were clustered in one clade. *MAM1/2, GSL-OH, IGMT2, GGP1, CYP81F2, CYP79B3*, and so forth indicated that multiple homologous genes might play similar roles in Chinese kale. However, there were also many homologous genes with varying gene expression models, such as *MAM3, AAO4*, and *STb*; similar results were also found in *B. juncea* (Meenu et al., 2015) and *B. rapa* (Zhang et al., 2015). Particularly, for the transcript factors, most of the homologous genes had different expression models (Figure 5B), which indicated that different homologous genes of transcript factors might play different roles in Chinese kale. Analysis of the genes using the expression data had also led to findings of the functional diversification and action pattern of different members of a gene family. Because of the duplicated nature of genes in *Brassica* plants, their expression was varied and complicated relative to those in *Arabidopsis*, and more experimentation is needed to ascertain expression models of these glucosinolate metabolism genes in Chinese kale and other *Brassica* plants.

## Conclusions

For the first time, this study has sequenced all the transcripts and comprehensively characterized the Chinese kale transcriptome. The study analyzed expression patterns in 11 different tissues that provided a general insight into the global view of the Chinese kale transcriptome. Most notably, the unigenes involved in glucosinolate metabolism were extracted and their expression patterns in different tissues were analyzed. The latter indicated that the roots, petiole and senescent leaves of the Chinese kale might be the main tissues where biosynthesis of glucosinolate takes place. Although, the molecular functions of the glucosinolate metabolism genes still remain largely unknown, the transcriptome and expression dataset analysis provided valuable information that can help to understand the molecular mechanisms underlying glucosinolate biosynthesis. The characteristic sequences studied by us will also serve as a gene resource for breeding programs of Chinese kale and other related plants.

## Author contributions

CC and BC contributed to designing the experiments. CC, SW, GC, and HC performed the experiments, collected, and analyzed the data. CC, JL, and SW contributed to data interpretation and preparation of the manuscript. All authors read and approved the final manuscript.

### Conflict of interest statement

The authors declare that the research was conducted in the absence of any commercial or financial relationships that could be construed as a potential conflict of interest.

## Supplementary material

Supplementary Table 1 **Primers used for qRT-PCR analysis**.

Supplementary Table 2 **Details of the reads used for *de novo* transcriptome assembly**.

Supplementary Table 3 **Statistics of unigene assemblies of whole plant and 11 tissues**.

Supplementary Table 4 **Summary statistics of functional annotation for Chinese kale unigenes in public databases**.

Supplementary Table 5 **The numbers of genes specifically expressed in one or two of the 11 tissues studied**.

Supplementary Data Sheet 1 **Expressions level of partial unigenes in different tissues**. The gene expression level is presented in FPKM (fragments per kilobase of exon per million fragments mapped).

Supplementary Data Sheet 2 **Identification of genes likely involved in glucosinolates metabolism and regulation in Chinese kale**.

Supplementary Image 1 **Different tissues used in RNA-seq analysis**. FB, flower buds; YL, young leaf; ML, mature leaf; SL, senescent leaf; LV, leaf vein; Pe, petiole; YB, young bolting stem flesh; MB, middle bolting stem flesh; BS, bolting stem skin; CS, combining site; Ro, roots.

Supplementary Image 2 **Summary statistics of functional annotation for Chinese kale unigenes in public databases. (A)** Statistics of unigenes annotated by different database; **(B)** Distribution of unigenes matched to homology in different species.

Supplementary Image 3 **Categorization of gene ontology annotation at level 2 of all the unigenes obtained from the whole plant (11 tissues)**.

Supplementary Image 4 **Enrichment of unigene GO terms in different tissues**. The number of unigene GO terms categorized into each functional subcategory is present in the percentage of GO terms for that subcategory out of the total GO terms that the unigenes assigned to each tissue (z-axis). The enrichments of the unigenes categorized into each subcategory of different tissues were estimated by a χ^2^ test using the percentage of unigenes in the subcategory of the whole plant as the expected. ^*^a significance level of *P* ≤ 0.05; ^**^a significance level of *P* ≤ 0.01.

Supplementary Image 5 **COG function distribution of unigenes obtained from the whole plant (11 tissues)**.
