# Supplementary material for: De novo Transcriptome Assembly of Chinese Kale and Global Expression Analysis of Genes Involved in Glucosinolate Metabolism in Multiple Tissues
Source: Front Plant Sci. 2017 Feb 8;8:92. doi: 10.3389/fpls.2017.00092 (PMC5296335; doi:10.3389/fpls.2017.00092)
Supplement: Supplementary Table 1 — Primers used for qRT-PCR analysis. [file Table1.DOC]

**Supplementary Table 1 | Primers used for qRT-PCR analysis**

| Gene name | Gene ID | Specific primers for RT-PCR | |
| --- | --- | --- | --- |
|  |  | Forward (5´-3´) | Reverse (5´-3´) |
| *MAM1/2* | Unigene_73909 | TTCAACCACATAAGCCCATA | GAGATTTTACAACCCCAACA |
| *IPMDH1* | Unigene_38232 | GTGGCAAAACTGTAGCGGGT | ACAAATGGGACAACAATGAG |
| *BCAT-4* | Unigene_55852 | TTCCGACCAGACCAAAACGC | GGACCACTACCAAACAAGAT |
| *CYP79F1* | Unigene_70138 | TTGTCCTAACCTTCCCTCGT | CCTCCTTGCTTACCTTCTCT |
| *CYP83A1* | Unigene_71114 | GGGAGGCAGTTTGTAGCGT | ATCATCATCGGGGTGGTGG |
| *CYP83B1* | Unigene_71455 | TATCTTTGCGTCTGCTATGG | GATGAAGTGAGGAATGTGGT |
| *SUR1* | Unigene_76549 | TCCTTGAGCCTATCACACAC | ACATAACTCCTGACCCTTCC |
| *STb* | Unigene_45871 | CCCTTCTCCTCTTCCTCTTC | TTTGTCTGGTTACGGTCCTT |
| *FMOGS-OX1* | Unigene_57901 | AAAGTATTCTCTCCCCCGA | GACCACCACCACCTATCAA |
| *IGMT2* | Unigene_71928 | GATGATGATAATGAGTTGGG | TTACGTGGTGTAGTTGGTAG |
| *Actin-2* |  | GAGGCTCCTCTTAACCCAAA | CAGAATCAAGCACAATACCG |

**Supplementary Table 2 | Details of the reads used for de novo transcriptome assembly**

| Source tissue | Clean Reads | Obtain Bases(G) | Q20(%) | Q30(%) | GC(%) |
| --- | --- | --- | --- | --- | --- |
| flower buds (FB) | 17,794,601 | 5.32 | 97.99 | 95.59 | 48.39 |
| young leaf (YL) | 21,364,577 | 6.39 | 98.22 | 95.94 | 48.59 |
| mature leaf (ML) | 18,648,136 | 5.57 | 98.06 | 95.67 | 48.96 |
| senescent leaf (SL) | 21,312,782 | 6.36 | 98.17 | 95.9 | 48.96 |
| leaf vein (LV) | 17,967,584 | 5.36 | 98.16 | 95.87 | 48.06 |
| petiole (Pe) | 21,522,897 | 6.43 | 98.1 | 95.65 | 47.98 |
| young bolting stem (YB) | 19,982,978 | 5.98 | 98.32 | 96.09 | 48.31 |
| middle bolting stem (MB) | 19,297,098 | 5.77 | 98.09 | 95.76 | 48.25 |
| bolting stem skin (BS) | 19,149,408 | 5.72 | 98.07 | 95.6 | 47.62 |
| combining sites(CS) | 18,084,261 | 5.4 | 98.03 | 95.61 | 48.67 |
| Root (Ro) | 21,083,182 | 6.3 | 98.12 | 95.81 | 48.24 |
| average | 19,655,227 | 5.87 | 98.12 | 95.77 | 48.36 |
| Total | 216,207,504 | 241 |  |  |  |

**Supplementary Table 3 | Statistics of unigene assemblies of whole plant and 11 tissues**

| Source tissue | No. of unigenes | Total length (nt) | N50 (nt) | Mean length (nt) | Total Mapped Reads |
| --- | --- | --- | --- | --- | --- |
| FB | 72136 | 54404790 | 1157 | 754 | 32509572 (98.42%) |
| YL | 98423 | 55380551 | 915 | 562 | 36812066 (92.89%) |
| ML | 49582 | 33782142 | 979 | 681 | 34970166 (98.66%) |
| SL | 66565 | 50594336 | 1191 | 760 | 39010230 (98.31%) |
| LV | 68029 | 53371635 | 1211 | 784 | 33975224 (98.61%) |
| Pe | 73636 | 56430602 | 1199 | 766 | 40510710 (97.58%) |
| YB | 64622 | 49776666 | 1190 | 770 | 36863382 (98.6%) |
| MB | 62381 | 48401038 | 1210 | 775 | 36215294 (98.52%) |
| BS | 75617 | 55587239 | 1121 | 735 | 35127110 (97.65%) |
| CS | 63715 | 49136290 | 1190 | 771 | 34274388 (98.4%) |
| Ro | 75219 | 57780438 | 1199 | 768 | 39684954 (98.14%) |
| whole plants | 98180 | 80525339 | 1240 | 820 |  |

**Supplementary Table 4 | Summary statistics of functional annotation for Chinese kale** unigenes in public databases

| Public protein database | No. of unigene hit | Percentage (%) |
| --- | --- | --- |
| NR | 78,900 | 80.36 |
| SwissProt | 58,063 | 59.14 |
| KEGG | 37,182 | 37.87 |
| GO | 30,272 | 30.83 |
| COG | 18,728 | 19,08 |
| Annotated in at least  one database | 80688 | 82.18 |
| Total | 98,180 | 100 |

**Supplementary Table 5 | The numbers of genes specifically expressed in one or two of the 11** tissues studied

| Tissues | BS | CS | FB | LV | MB | ML | Pe | Ro | SL | YL | YB |
| --- | --- | --- | --- | --- | --- | --- | --- | --- | --- | --- | --- |
| BS | **2269** | 95 | 317 | 160 | 157 | 34 | 2264 | 285 | 73 | 120 | 162 |
| CS |  | **1141** | 199 | 62 | 69 | 26 | 63 | 873 | 67 | 45 | 71 |
| FB |  |  | **4498** | 140 | 95 | 55 | 84 | 563 | 174 | 203 | 207 |
| LV |  |  |  | **1755** | 97 | 68 | 392 | 196 | 229 | 71 | 69 |
| MB |  |  |  |  | **685** | 22 | 51 | 137 | 60 | 18 | 389 |
| ML |  |  |  |  |  | **406** | 31 | 41 | 326 | 169 | 17 |
| Pe |  |  |  |  |  |  | **1740** | 158 | 89 | 71 | 46 |
| Ro |  |  |  |  |  |  |  | **3247** | 301 | 48 | 118 |
| SL |  |  |  |  |  |  |  |  | **1445** | 100 | 36 |
| YL  YB |  |  |  |  |  |  |  |  |  | **2720** | 37  **701** |
