# Supplementary figures and images for: De novo Transcriptome Assembly of Chinese Kale and Global Expression Analysis of Genes Involved in Glucosinolate Metabolism in Multiple Tissues
Source: Front Plant Sci. 2017 Feb 8;8:92. doi: 10.3389/fpls.2017.00092 (PMC5296335; doi:10.3389/fpls.2017.00092)

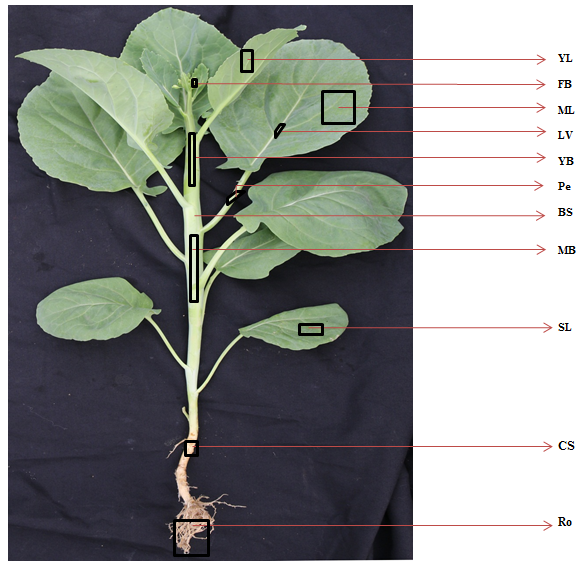

Supplement: Supplementary Image 1 — Different tissues used in RNA-seq analysis. FB, flower buds; YL, young leaf; ML, mature leaf; SL, senescent leaf; LV, leaf vein; Pe, petiole; YB, young bolting stem flesh; MB, middle bolting stem flesh; BS, bolting stem skin; CS, combining site; Ro, roots. [file Image1.TIF]

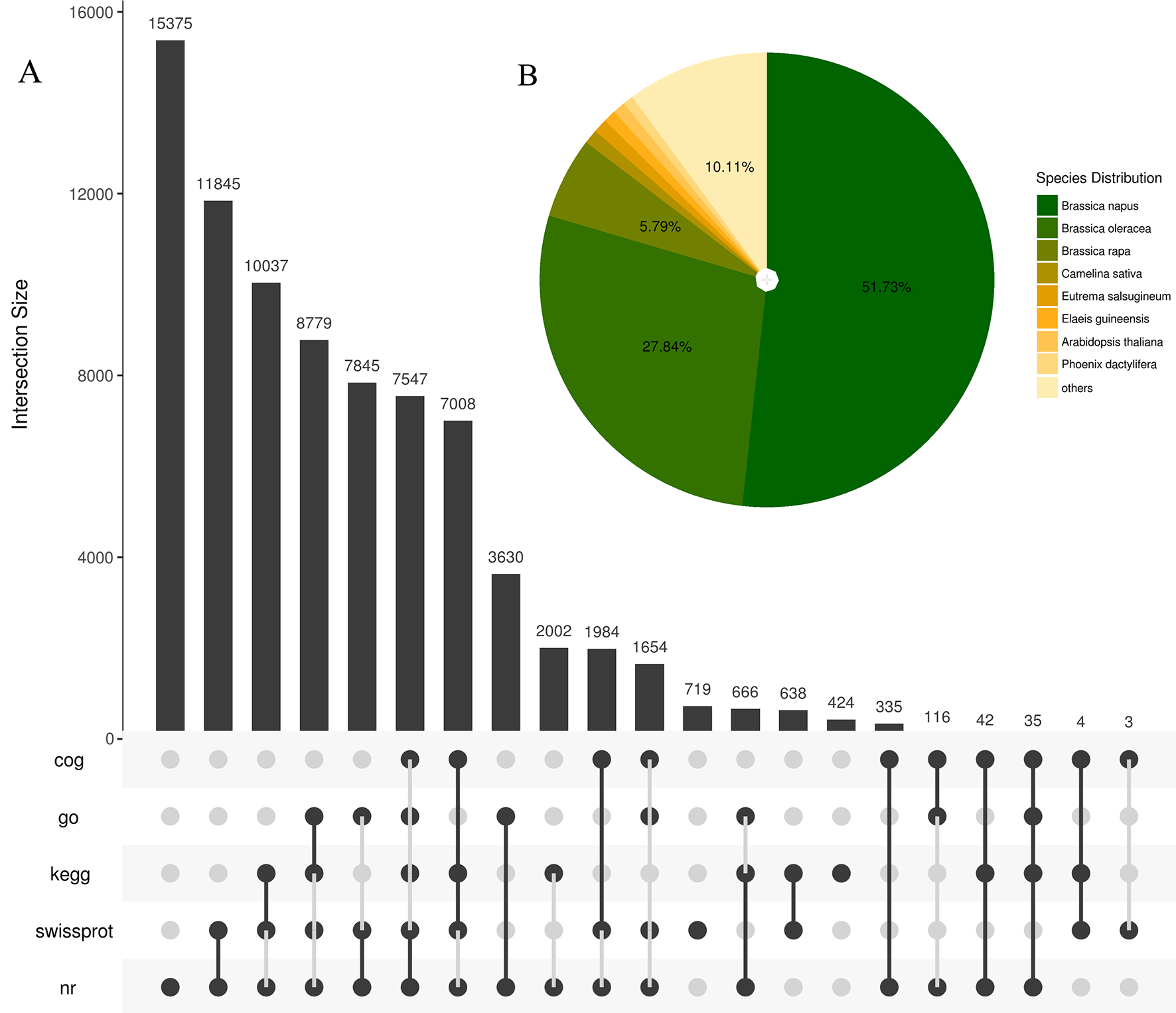

Supplement: Supplementary Image 2 — Summary statistics of functional annotation for Chinese kale unigenes in public databases. (A) Statistics of unigenes annotated by different database; (B) Distribution of unigenes matched to homology in different species. [file Image2.TIF]

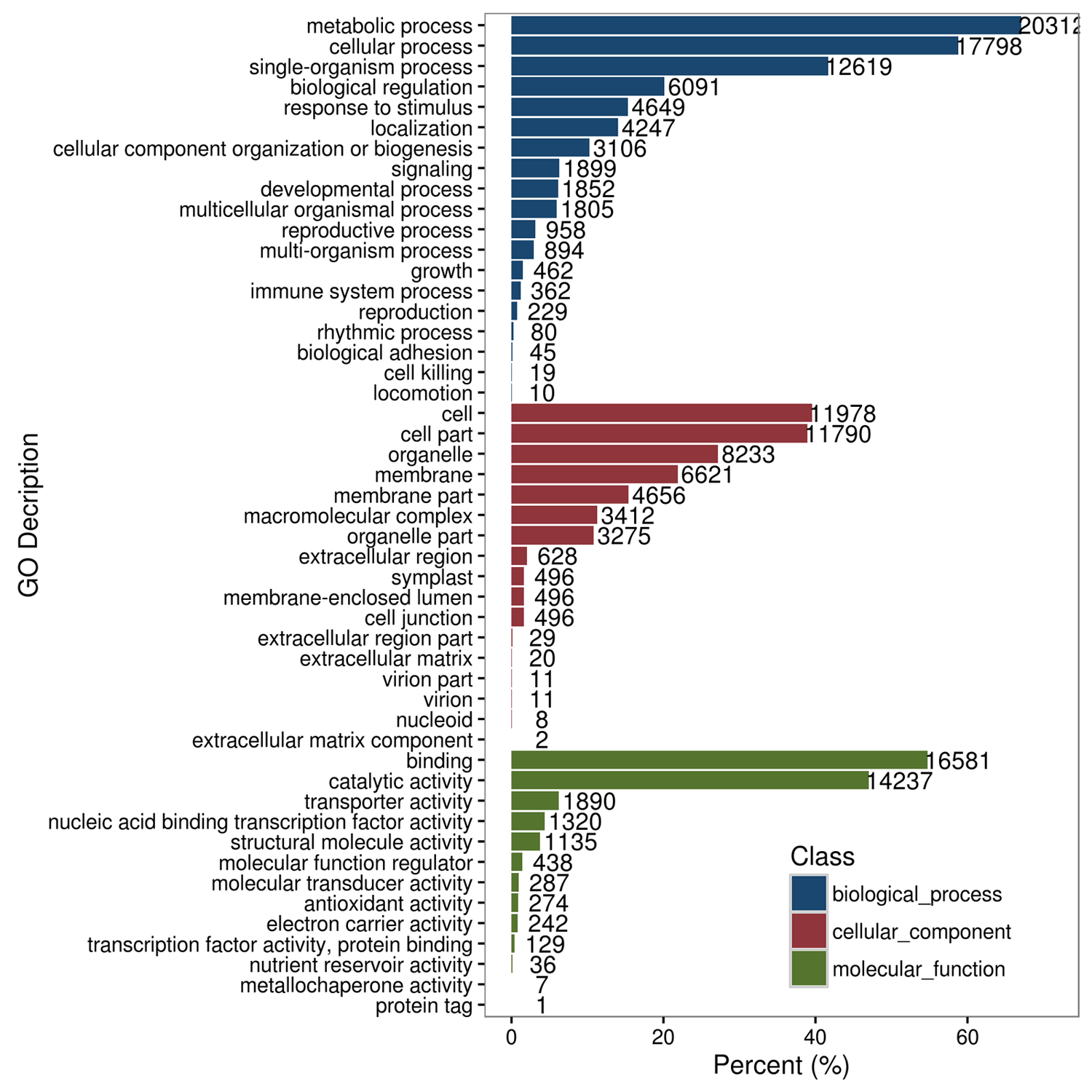

Supplement: Supplementary Image 3 — Categorization of gene ontology annotation at level 2 of all the unigenes obtained from the whole plant (11 tissues). [file Image3.TIFF]

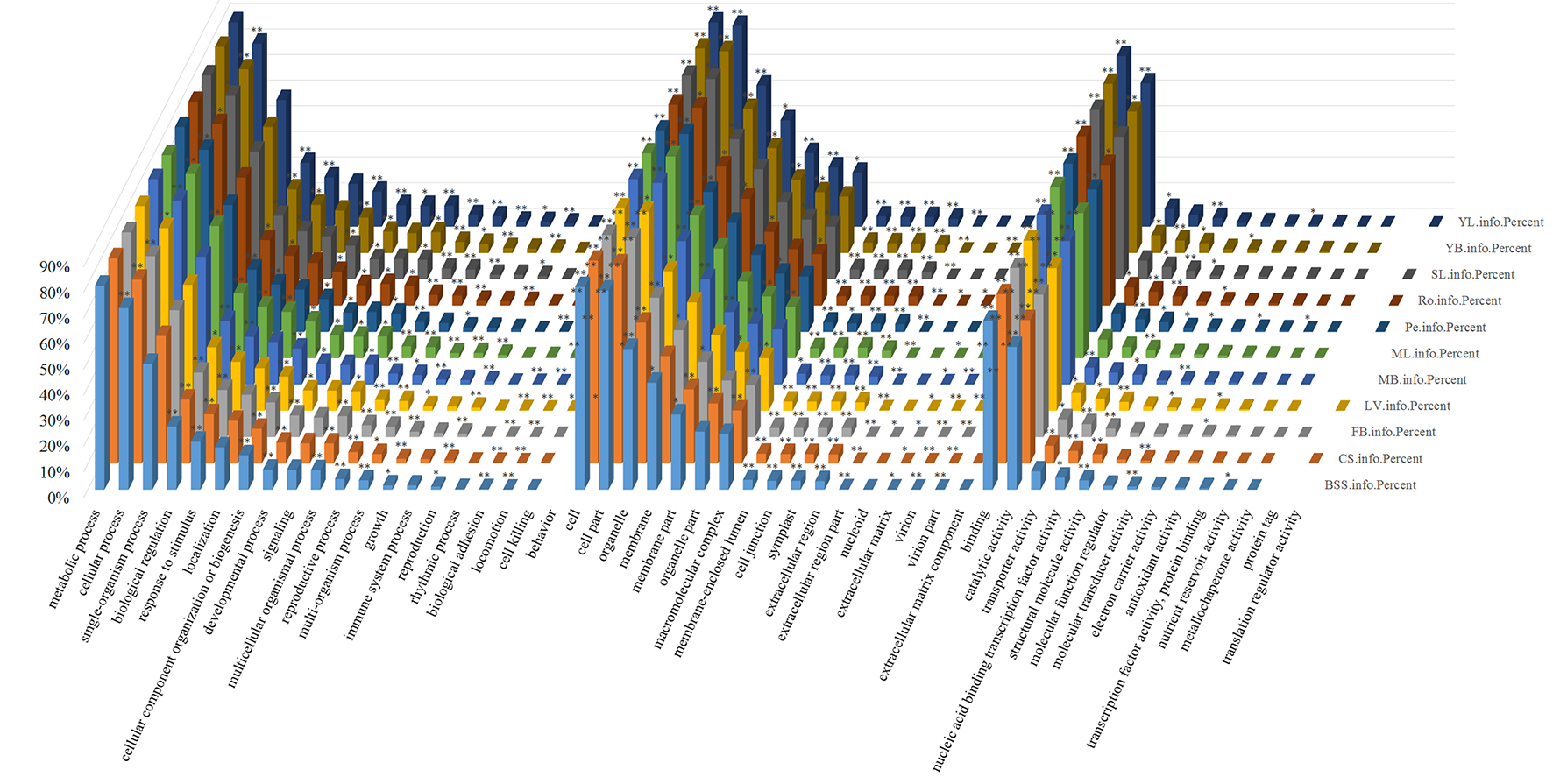

Supplement: Supplementary Image 4 — Enrichment of unigene GO terms in different tissues. The number of unigene GO terms categorized into each functional subcategory is present in the percentage of GO terms for that subcategory out of the total GO terms that the unigenes assigned to each tissue (z-axis). The enrichments of the unigenes categorized into each subcategory of different tissues were estimated by a χ2 test using the percentage of unigenes in the subcategory of the whole plant as the expected. *a significance level of P ≤ 0.05; **a significance level of P ≤ 0.01. [file Image4.TIF]

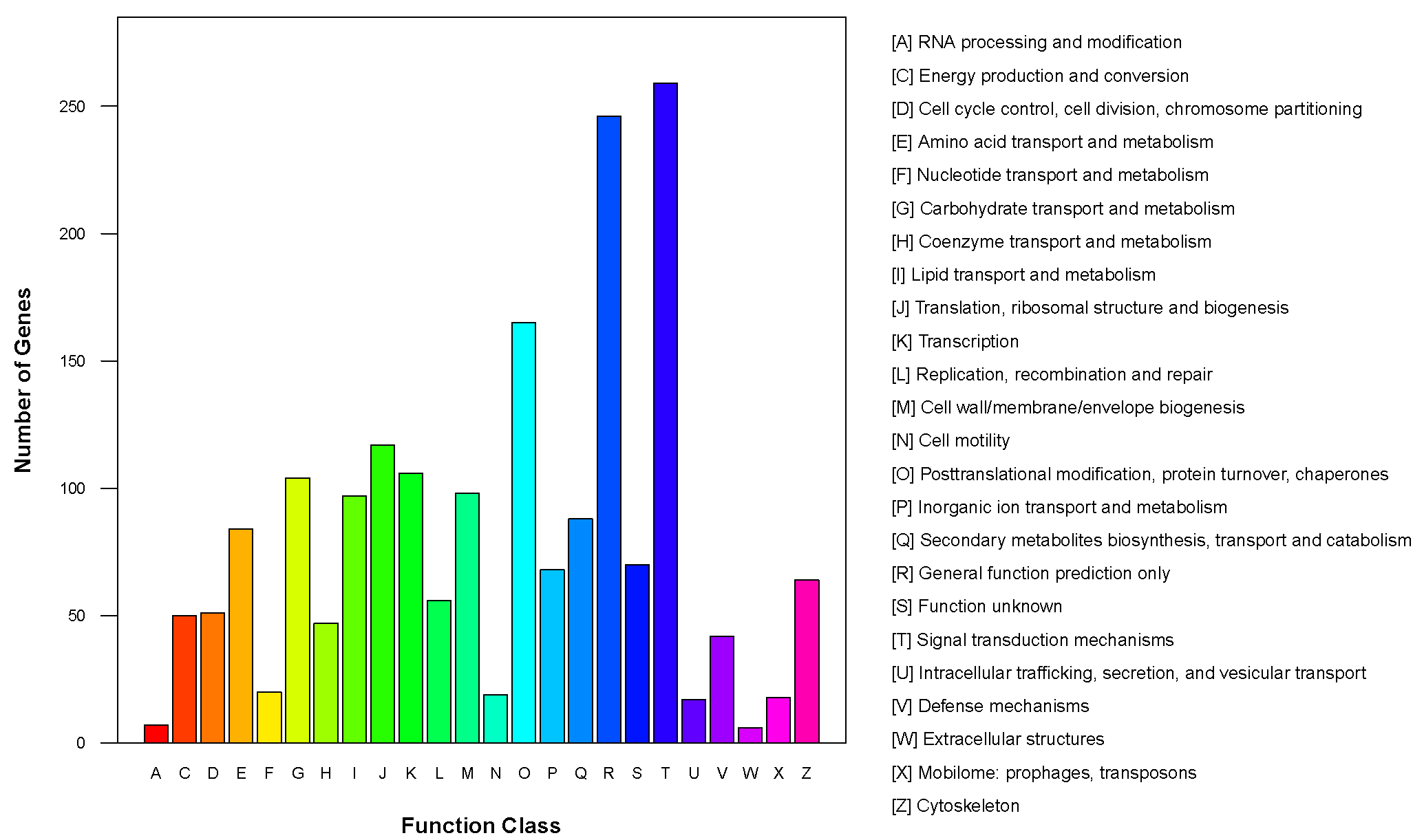

Supplement: Supplementary Image 5 — COG function distribution of unigenes obtained from the whole plant (11 tissues). [file Image5.TIFF]
